# Nicotine associated breast cancer in smokers is mediated through high level of EZH2 expression which can be reversed by methyltransferase inhibitor DZNepA

**DOI:** 10.1038/s41419-017-0224-z

**Published:** 2018-02-02

**Authors:** Kanchan Kumari, Biswajit Das, Amit Adhya, Sanjib Chaudhary, Shantibhusan Senapati, Sandip K. Mishra

**Affiliations:** 1 Cancer Biology Laboratory, Department of Gene Function and Regulation, Institute of Life Sciences, Bhubaneswar, Odisha India; 2 Utkal University, Bhubaneswar, Odisha India; 3 Tumor Microenvironment and Animal Models Laboratory, Department of Translational Research, Institute of Life Sciences, Bhubaneswar, Odisha India; 4 Manipal University, Manipal, Karnataka India; 5 Pathology Department, Hemalata Hospital, Chandrashekharpur Bhubaneswar, Odisha India; 6 University of Nebraska Medical Center, Nebraska, NE USA

## Abstract

Recent studies show substantial growth-promoting properties of nicotine (NIC) in cancer, which is a combined outcome of genetic and epigenetic alterations. However, the role of epigenetic modifiers in response to NIC in breast cancer is less studied. In the present study, for the first time we have shown NIC-induced enhanced EZH2 expression. Six pairs of smoking-associated breast cancer patient tissues were analyzed. Samples from smoking breast cancer patients showed distinguished enhanced EZH2 expression in comparison to non-smoking ones. The upregulation in EZH2, which is due to NIC, was further confirmed in breast carcinoma cell lines using 10 µM NIC, 1 µM DZNepA, and EZH2si. The upregulation of EZH2 was concomitant with upregulation in Myc and α9-nAChR. The xenograft of breast cancer cells in BALB/c nude mice in the presence or absence of NIC showed significantly higher tumor uptake in the NIC injected group, which clearly demonstrates the effect of NIC in breast cancer progression. Interestingly, DZNepA considerably suppressed the NIC-mediated tumor growth. CHIP-qPCR assay confirmed the increased Myc enrichment on EZH2 promoter upon NIC treatment, thereby strengthening our findings that there exists an association between NIC, Myc, and EZH2. Overall, the present study identifies a strong association between NIC and EZH2 particularly in the progression of breast cancer in smokers through a novel axis involving nAChR and Myc. Moreover, the findings provide preliminary evidence suggesting potential of high level of EZH2 expression as a prognostic marker in smoking-associated breast cancer.

## Introduction

Breast cancer is the most common cancer in females. According to the cancer facts and figures provided by the American Cancer Society for the year 2017^1^, there are 40,610 and 252,710 number of estimated deaths and new cases respectively for breast cancer in females. It stands at third position in mortality and second for new cases in USA. Regardless of great achievements in the area of cancer research, there is a big question about the causal of the disease. Exploration of the research findings broadly categorizes the causal factors for breast cancer into genetic and environmental. In-between these two factors there exists another factor called epigenetics that play a significant role in disease progression. Among the list of factors that are preventable, smoking is the one, which is being increasingly prevalent in females round the world including developing countries like India^2^. Smoking is consistently linked to increased breast cancer risk^3–5^. Cigarette smoke consists of more than hundreds of constituents^6^ among which nicotine (NIC) has been widely studied for its effects on neurons^7^. NIC is studied for its unfavorable effects on breast cancer too^8–10^. Association of active and passive smoking with breast cancer is reported^11, 12^. Nicotinic acetylcholine receptors (nAChR) are the receptors through which NIC functions and are upregulated by it^13^. During NIC-induced transformation of normal breast epithelial cells, overexpression and activation of α9-nAChR is reported^14, 15^. Research in this area has shown that NIC makes cancer cells more aggressive^16^.

Covalent modifications of histone proteins play a fundamental role in structure and function of chromatin. One such modification that is central to gene regulation is histone methylation^17^, which is carried out by histone methyltransferases. Polycomb group protein enhancer of zeste homolog 2 (EZH2) is a histone methyltransferase enzyme that tri-methylates histone H3 at lysine 27 leading to gene repression. Remarkable oncogenic role of EZH2 is reported in several studies^18, 19^. EZH2 is upregulated in breast carcinoma cells and is associated with aggressiveness of the disease^20^.

Here in this study, we showed close association of EZH2 and NIC-induced increased breast cancer progression. Samples from smoking and never-smoked breast cancer patients, cell lines and xenografts were assessed for EZH2 expression level and its functional role in response to NIC in breast cancer pathogenesis. Using EZH2 inhibitor DZNepA and EZH2si in NIC-treated breast cancer cells, involvement of EZH2 in NIC-mediated aggressiveness of the disease was evaluated. An increased tumor uptake was observed in the xenograft mice model upon NIC treatment. Also, our in vivo studies demonstrated the efficacy of DZNepA in reducing the tumor burden. Using Bupropion, an NIC antagonist, we validated the NIC-induced Myc-mediated EZH2 expression. Increased fold enrichment of Myc on EZH2 promoter upon NIC treatment as obtained by CHIP-qPCR strengthened the involvement of Myc in NIC-mediated enhanced EZH2 expression. Overall, we show that NIC exposure either through smoking in breast cancer patients or upon direct treatment in vitro and in vivo resulted into an increased EZH2 expression in breast cancer cells.

## Results

### Breast cancer patient samples from smoking individuals harbor enhanced EZH2 expression

**Fig. 1:**
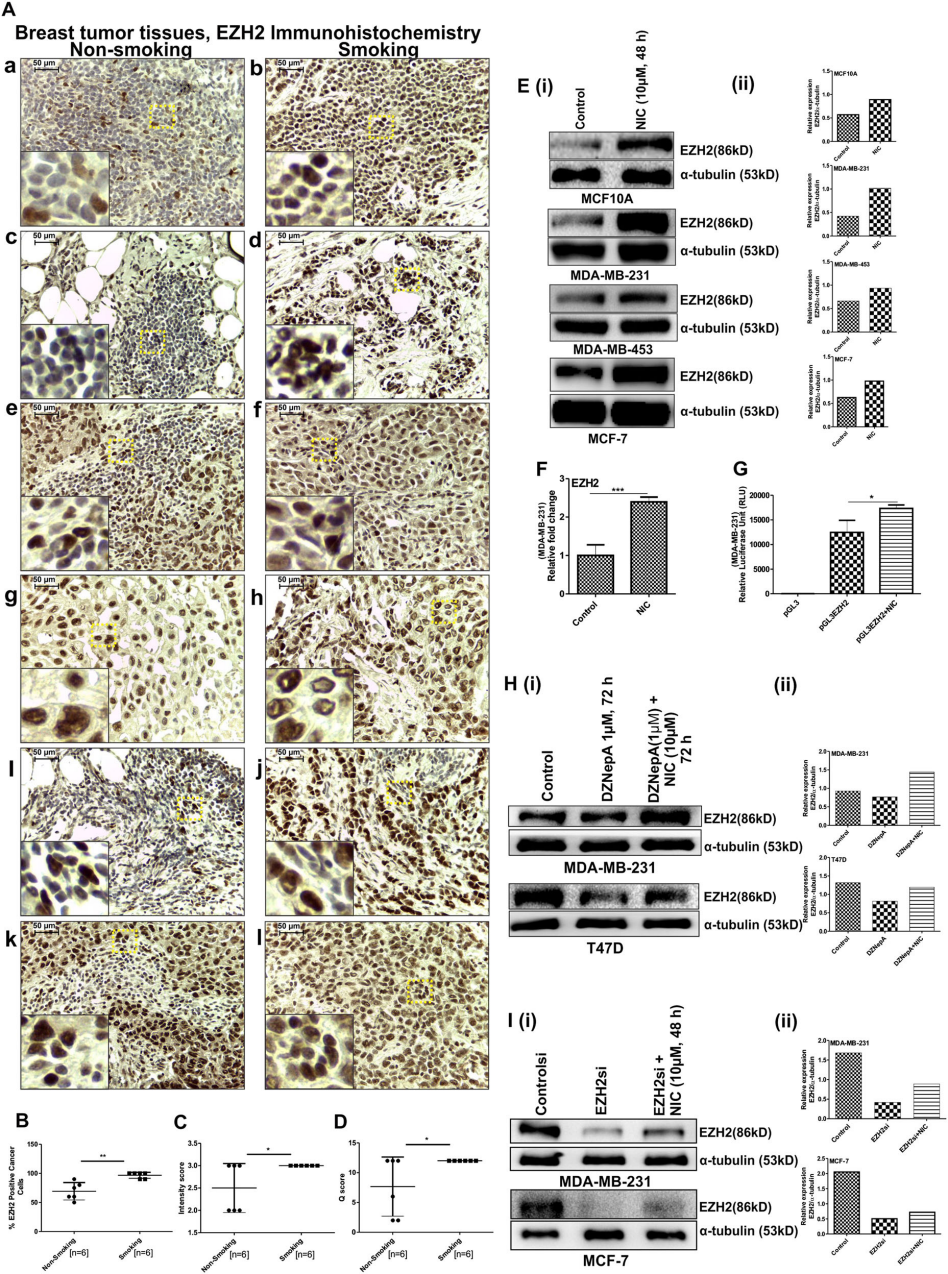
NIC exposure either through smoking in breast cancer patients or upon direct treatment in breast cancer cells results into increased EZH2 expression. **a** Immunohistochemical stained breast cancer patient samples from smoking and non-smoking individuals showing the difference in EZH2 expression. Insets show 10 times digitally magnified pictures of images captured with ×40 objective. **b** Scatter plot shows the percentage of EZH2-positive cancer cells in both the groups. **c** Difference in the intensity score of EZH2 expression in two groups is depicted in the graph. **d** Graph represents the *Q* score for EZH2 expression in tissue sections of smoking and never-smoked breast cancer patients. **e** (i) Western blot shows upregulated EZH2 expression upon NIC treatment in breast cancer cell lines including normal breast epithelial MCF-10A cells. **e** (ii) Quantification of western blot was done using software ImageJ. **f** Graph displays the relative fold change of EZH2 expression at mRNA level between control and NIC-treated MDA-MB-231 cells. **g** Increased EZH2 promoter activity was observed in luciferase assay upon NIC treatment in cancer cells. **h** (i), **i** (i) Immunoblot showing the depleted EZH2 protein level in MDA-MB-231 breast cancer cells upon DZNepA treatment and after EZH2si transfection, respectively, which was recovered following NIC treatment. **h** (ii), **i** (ii) Graphs showing normalized EZH2 expression calculated with respect to α-tubulin. Graphs are plotted with SD, which is calculated from indicated number of samples used for the study. Two-tailed paired Student's *t*-test and one-way ANOVA was used for statistical analysis. *P < 0.05, **P < 0.005, ***P < 0.001. Scale bar 50 µm

Six pairs of breast cancer tissue (biopsy) from smokers or non-smokers were included in the study. All the cases were carefully selected and compared based on age of the breast cancer patients, the minimum stage of the disease based on grade and histological similarity of the tumor as EZH2 expression is associated with aggressiveness of the disease. Pairs of never-smoked and smoking-associated patient samples 1Aa and 1Ab, 1Ag and 1Ah, and 1Ak and 1Al belonging to similar stage of disease and tumor grade displayed increased EZH2 expression in smoking ones. In one of the pair of samples, non-smoking sample 1Ae is from more advanced stage (metastatic adenocarcinoma with a higher tumor grade 3) whereas the smoking tumor tissue sample 1Af is from a comparatively less aggressive breast cancer (non-metastatic and tumor grade 2), but EZH2 expression is still significantly high in cancer tissue from smoking individual. In another pair of samples 1Ai and 1Aj, both are of stage IIIC; the non-metastatic breast tumor associated with smoking harbor higher EZH2 expression in comparison to non-smoking counterpart, which is from more aggressive, i.e. metastatic breast tumor. Although higher EZH2 expression harboring smoking-associated sample 1Ad is from higher-grade tumor, the sample 1Ac from never-smoked individual is a metastatic adenocarcinoma. The overall analysis of clinical samples for EZH2 expression level among smokers and non-smokers led us a hypothesis that EZH2 might have some role in NIC-mediated breast cancer progression (Fig. 1a–d).

### NIC treatment results into enhanced EZH2 expression in breast carcinoma cells and abrogates the effect of DZNepA or EZH2si

The association of NIC with a high level of EZH2 was confirmed in vitro in both cancerous and non-cancerous breast cancer cell lines. To validate our hypothesis that EZH2 plays a significant role in NIC-mediated increased breast cancer progression without limiting to specific cell line, we included one normal breast epithelial cell line MCF-10A, two estrogen receptor positive cell lines T47D and MCF-7, and two aggressive estrogen receptor negative breast cancer cell lines MDA-MB-231 and MDA-MB-453 in the study where we found similar kind of results. NIC treatment on normal breast epithelial cells and breast carcinoma cells resulted in the overexpression of EZH2 (Fig. 1e) as detected by western blot assay. The high expression level of EZH2 was also confirmed by quantitative reverse transcriptase-polymerase chain reaction (qRT PCR) (Fig. 1f). The upregulation of EZH2 was associated with strong promoter activity upon NIC treatment as revealed from luciferase assay (Fig. 1g). As already reported, DZNepA treatment depletes EZH2 through increased proteasome-mediated degradation or accumulation of strong product inhibitor *S*-adenosyl homocysteine^21^; we checked the effect of NIC on EZH2 expression post-DZNepA treatment where NIC was able to abrogate the effect of DZNepA (Fig. 1h). At the same time, NIC inhibited the effect of EZH2si on EZH2 expression. (Fig. 1i).

### Inhibition of EZH2 by DZNepA or EZH2si reduces NIC-induced breast cancer progression

**Fig. 2:**
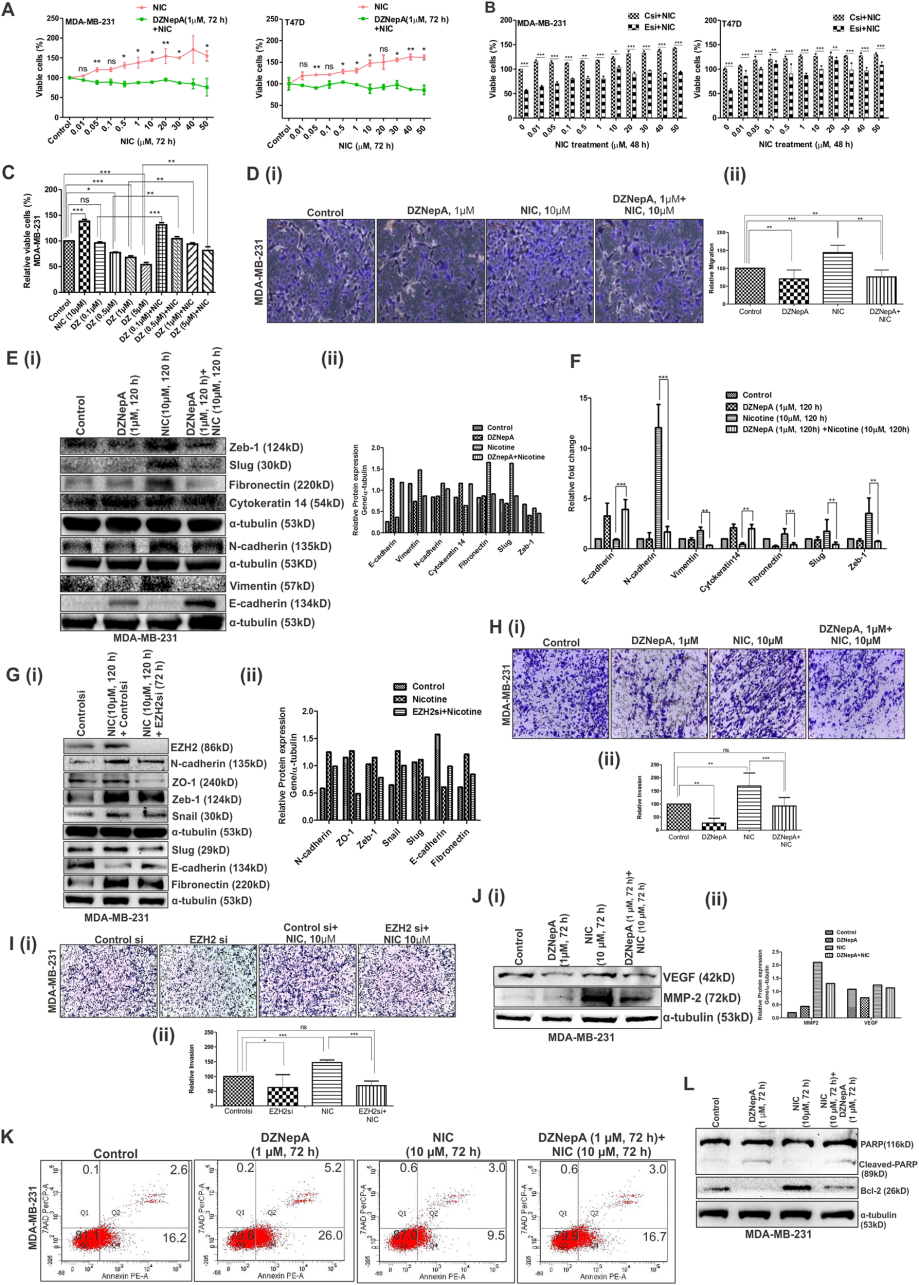
Transfection of EZH2si or treatment of DZNepA in NIC-treated breast carcinoma cells led to reduced breast cancer progression. **a** MTT assay shows the antagonistic effect of DZNepA on NIC-mediated increased number of viable cells in MDA-MB-231 and T47D breast cancer cells. **b** Graph illustrates number of viable cells upon EZH2si transfection in NIC-treated cells. **c** Effect of varying concentrations of DZNepA or co-treatment of NIC and DZNepA on number of viable cells after 72 h of treatment. **d** (i) Picture showing ×20 images of transwell migration assay performed to study the effect of DZNepA on NIC-mediated increased cell migration. **d** (ii) Graph depicts the relative percent of migration in all four experimental groups in comparison to control groups. **e** (i), **g** (i) Expression of proteins involved in epithelial to mesenchymal transition (EMT) upon NIC/DZNepA/EZH2si treatment/transfection. **e** (ii), **g** (ii) Normalized protein expression as observed in western blots. **f** qRT PCR shows the expression of EMT-related genes at mRNA level. **h** (i), **i** (i) Representative pictures (×20) of invasion assay demonstrates the inhibitory effect of DZNepA and EZH2si, respectively, on NIC-induced increased invasion of cancerous cells. **h** (ii), **i** (ii) Graph shows the relative percent of invasion compared to control in the experimental groups for experiments done in triplicate. **j** (i) Immunoblot displaying the effect of DZNepA on NIC-induced increased MMP-2 and VEGF expression. **j** (ii) Bar diagram shows normalized protein expression. **k** The effect of NIC on DZNepA-mediated increased apoptosis in MDA-MB-231 cells is evident from the flow cytometry data. **l** Difference in the expression of Bcl-2 and Cleaved-PARP in different experimental groups is shown in the western blot. Graphs are plotted with SD, which is calculated from three independent experiments. Two-tailed paired Student's *t*-test, one-way and two-way ANOVA were used for statistical analysis for experiments done in triplicate. *P < 0.05,**P < 0.005, ***P < 0.001

Increasing concentration of NIC in MDA-MD-231 and T47D cell lines showed significant increase in the number of viable cells. Simultaneous treatment of 1 µM DZNepA in cells treated with varying concentrations of NIC resulted in notable reduction in the number of viable cells (Fig. 2a). Similarly, in cells treated with varying NIC concentrations, a notable decrease in the number of viable cells was observed upon EZH2si transfection (Fig. 2b). MTT assay performed with varying concentrations of DZNepA significantly reduced the number of viable cells. In comparison to only DZNepA-treated cells, a subsequent significant increase in the percentage of viable cells was found in cells co-treated with 10 µM NIC and respective increasing concentration of DZNepA, which indicated the inhibitory effect of NIC on DZNepA (Fig. 2c). Increased cell migration upon NIC treatment was found to be inhibited by DZNepA (Fig. 2d). Further, a notable increase in mesenchymal markers and a decrease in epithelial cell markers in NIC-treated cells was found to be reversed in DZNepA and NIC co-treated cells at both protein and mRNA level (Fig. 2e, f). Role of EZH2 in epithelial to mesenchymal transition (EMT) is well known. To investigate the role of NIC in modulating EMT markers by elevating the level of EZH2 in cancer cells, the cancer cells were first treated with NIC for 72 h and then the NIC-treated cells were transfected with controlsi or EZH2si (in absence of NIC). After 24 h of transfection, cells were again treated with NIC for another 48 h. Immunoblot showed increased EZH2 level and altered expression of proteins involved in EMT in NIC-treated cells, which was inhibited upon EZH2 knocked-down, thus signifying the fact that NIC modulates the EMT markers in an EZH2 dependent manner (Fig. 2g). Also, NIC-induced increased cell invasion was checked upon DZNepA treatment (Fig. 2h) and EZH2si transfection (Fig. 2i). Apart from the role of VEGF in angiogenesis, it also increases cancer cell growth, migration, and invasion^22^. Also, it is involved in key aspects of tumorigenesis such as tumor initiation^23^. Cancer cell growth and invasion is affected by altered expression of matrix metalloproteinase 2^24^. Further, to validate the previously reported NIC-mediated increased levels of VEGF^25^ and MMP-2^26^, we checked their expression in MDA-MB-231 upon NIC/DZNepA treatment. A reduced expression of VEGF and MMP-2 expression was observed in DZNepA and NIC co-treated cells in comparison to only NIC-treated cells, which emphasizes on the role of NIC on cell invasion property through EZH2 (Fig. 2j). DZNepA is reported to induce apoptosis in cancer cells^21^; on the other hand, NIC provides survival advantage to the cells by making them resistant to apoptosis. More than 10% reduction in early apoptotic cells were observed in cells simultaneously treated with NIC and DZNepA (Fig. 2k). Apoptosis is regulated in part by Bcl-2 genes that regulate cell survival^27^. Reduced apoptosis and neuroprotective effect of NIC is marked by reduced cleavage of PARP-1^28, 29^. NIC treatment resulted into a significant increase in Bcl-2 expression that reduced upon DZNepA treatment. Additionally, reduced PARP cleavage in cells co-treated with NIC and DZNepA in comparison to only DZNepA-treated cells corroborated the protective effect of NIC on DZNepA-induced apoptosis (Fig. 2l). These data showed the effect of DZNepA and EZH2si on NIC-induced enhanced breast cancer cell progression and thus indicated the direct involvement of EZH2.

### NIC treatment led to increased tumor uptake in xenograft nude mice model and upon DZNepA treatment, NIC-induced increased tumor growth was significantly reduced

**Fig. 3:**
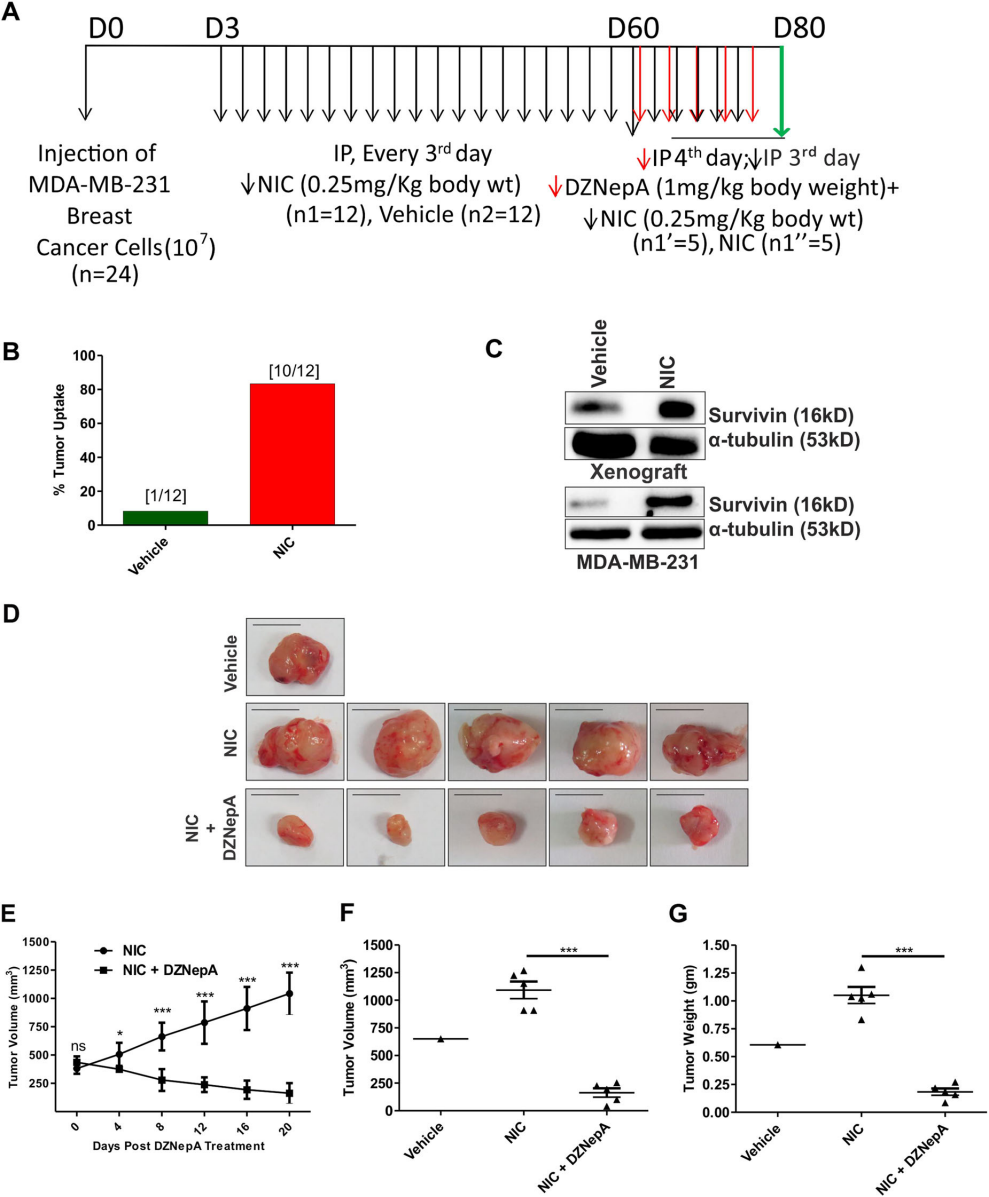
Increased tumor uptake was found in NIC-treated mice and DZNepA significantly reduced the enhanced tumor growth in response to NIC treatment. **a** Diagram depicts the overall xenograft study. **b** Graph showing percent tumor uptake in vehicle and NIC-treated group of mice. **c** Immunoblot shows survivin expression in response to nicotine exposure in xenograft and MDA-MB-231 breast cancer cells. **d** Picture of tumors collected at the end of the experiment in the three experimental groups. **e** Graph demonstrates the effect of DZNepA on NIC influenced tumor growth. **f**, **g** Difference in tumor volume and weight in three experimental groups are illustrated in the graph respectively. Black and red arrow indicates IP injection of NIC and DZNepA, respectively. Green arrow indicates the day of termination of experiment. As a reference, the ruler was used while capturing the digital image of individual tumor and scale bar of 1 cm is used as a reference for tumor size. Two-tailed paired Student's *t*-test was used for statistical analysis. *P < 0.05, ***P < 0.001

To understand the role of NIC in increasing the breast cancer risk, the xenograft nude mice model was used. Twenty-four mice were involved in the study for 11 weeks (Fig. 3a). We considered 100 mm^3^ tumors size as cut-off volume for a well-established tumor. After 60 days of treatment, among 12 vehicle-treated mice, only 1 developed and established the tumor. However, out of 12 NIC-treated mice, 10 mice developed the tumor, which was growing well. This suggested that NIC increases the tumor uptake by 75% (Fig. 3b) and thus might be a strong risk factor for breast cancer. Role of survivin in malignant transformation of normal human bronchial epithelial cells is previously reported^30^. To validate that NIC provides survival advantage to cancer cells^28, 31^, we checked the expression of survivin upon NIC treatment both in vitro and in vivo. In response to NIC treatment, expression of survivin was upregulated in both xenograft and MDA-MB-231 breast cancer cells (Fig. 3c), thus supporting the proposal that NIC provides survival benefit to the cancer cells which led to increased tumor uptake in mice model. Upon DZNepA treatment in NIC-treated mice, the mean tumor volume was reduced by 74% (Fig. 3f). However, the xenografts of NIC-treated mice grew exponentially.

### Enhanced expression of EZH2 and Ki67 in xenografts from NIC-treated mice was reduced upon DZNepA treatment

**Fig. 4:**
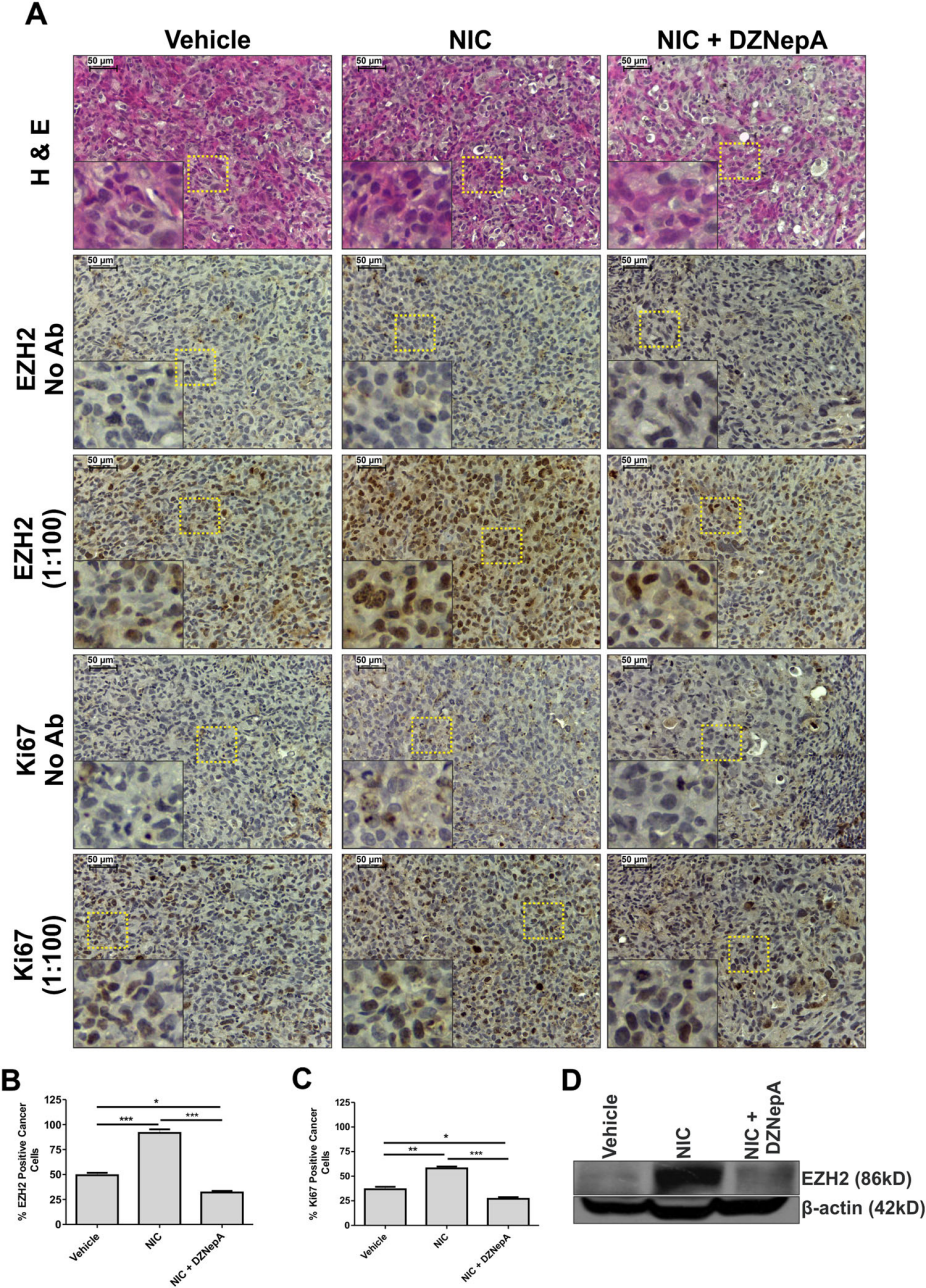
Tumor tissue sections of NIC-treated mice showed enhanced expression of both EZH2 and Ki67. **a** Immunohistochemistry in tissue sections prepared from tumors collected from vehicle, NIC, and NIC and DZNepA xenografts shows the effect of drugs on the expression of EZH2 and Ki67. Insets show 10 times digitally magnified pictures of images captured with ×40 objective. **b**, **c** Graph depicts the percentage of EZH2- and Ki67-positive cancer cells in the three groups. **d** Western blot shows the EZH2 expression level in tumor of three groups. One-way ANOVA was used for statistical analysis *P < 0.05, **P < 0.005, ***P < 0.001. Scale bar 50 µm

Similar to the results observed in smoking patient samples, immunohistochemistry in tumor sections of NIC-treated (0.25 mg/kg body weight, 80 days) mice showed 30% enhanced EZH2 expression (Fig. 4a, b) . At the same time, expression of proliferation marker Ki67 was upregulated by 22.5% in NIC-treated tumor samples (Fig. 4a, c). Upon DZNepA treatment (1 mg/kg body weight, 20 days), expression of EZH2 was reduced by 48% and a reduction of 36.5% was observed in the expression of Ki67, which clearly indicated the potential of DZNepA in reducing the tumor burden. A similar trend of EZH2 expression was also observed in protein extracted from vehicle, NIC, and NIC and DZNepA co-treated xenografts by western blot assay (Fig. 4d).

### NIC dependent enhanced EZH2 expression is induced by increased expression of Myc in response to NIC exposure

**Fig. 5:**
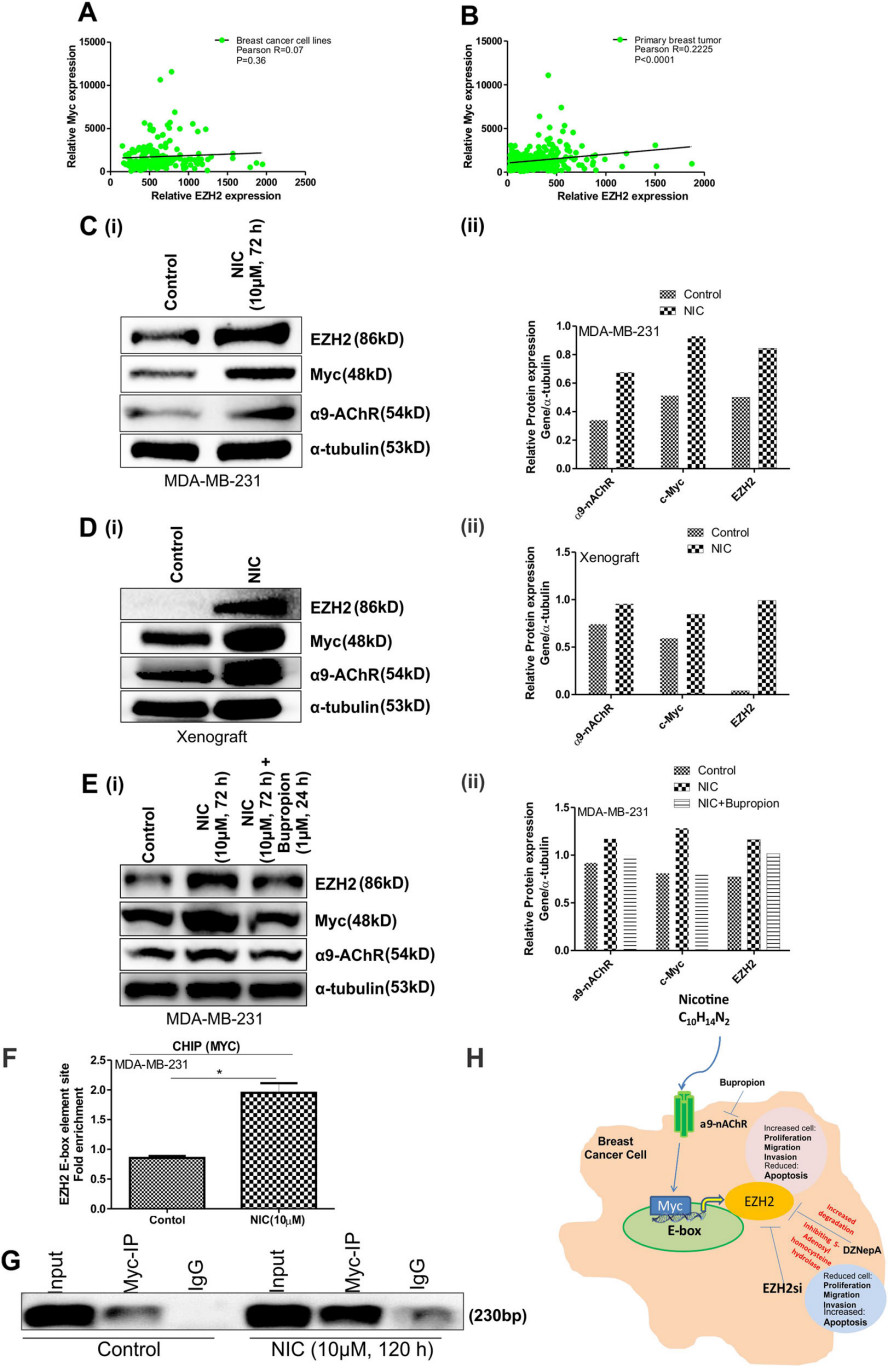
NIC dependent increased EZH2 expression is a result of upregulated Myc in response to NIC. **a**, **b** Graph depicts correlation between EZH2 and Myc in breast cancer cell lines and primary breast tumor respectively. **c** (i), **d** (i) Immunoblot shows NIC-mediated altered expression of α9-nAChR, Myc, and EZH2 in breast cancer cells and xenograft respectively. **c** (ii) and **d** (ii) Densitometry analysis of the respective immunoblot is displayed in the graph. **e** (i) and (ii) Effect of Bupropion (1 µM) treatment on NIC-mediated response is displayed by the western blot and densitometry graph, respectively. **f** Fold enrichment of Myc on EZH2 promoter in control and NIC-treated MDA-MB-231 cells is depicted in the graph as observed by CHIP-qPCR assay. **g** Corresponding agarose gel picture of the CHIP-qPCR product showing the expression of the EZH2 (E-box element containing) promoter region amplified with immunoprecipitated-Myc as a template in both control and NIC-treated cells. **h** Diagram depicts the proposed pathway involved in EZH2-mediated increased breast cancer progression in response to NIC. Graphs are plotted with SD, which is calculated from three independent experiments. Two-tailed paired Student *t*-test was used for statistical analysis. *P < 0.05

Myc is a transcription factor, which positively regulates EZH2 by directly binding on the E-box regulatory promoter region^32^. Positive correlation exists between EZH2 and Myc in breast cancer cells (Fig. 5a) as well as in primary breast tumor (Fig. 5b) as computed from the MERAV expression datasets^33^. In response to NIC, an increased expression of α9-nAChR, Myc, and EZH2 was observed in vitro (Fig. 5c) and in vivo (Fig. 5d). Upon treatment with the NIC antagonist Bupropion, the effect of NIC was reversed and reduced expression of EZH2 and Myc was observed in MDA-MB-231 cells (Fig. 5e). CHIP-qPCR showed about two-fold enrichment of Myc at E-box element (CACGTG) on EZH2 promoter in NIC-treated MDA-MB-231 cells (Fig. 5f, g). These data suggested the possible mechanism behind the enhanced EZH2 expression in response to NIC in breast cancer and thus in increased disease progression (Fig. 5h).

## Discussion

Tumor-promoting activity of NIC is reported in various types of cancers. Upon activation by NIC, nAChR activate several growth stimulating signaling pathways. In breast cancer, activation of specific nAChRα-9 is reported during transformation of normal breast epithelial cells. Although NIC is studied for its effect on non-neuronal cells, such as cancer cells, association of NIC-dependent epigenetic alteration with cancer pathogenesis is not known. In this study, we demonstrated that NIC-induced increased breast cancer progression is mediated through EZH2 and enhanced EZH2 expression in response to NIC is primarily through Myc upregulation. As NIC is one of the constituents of cigarette smoke, degree of NIC exposure in the smoking individuals and the corresponding effect on EZH2 expression is debatable. However, in in vitro cell culture, enhanced expression of EZH2 was detected even at nanomolar concentrations within 24 h of NIC treatment (Figure S2). Our luciferase assay data (Fig. 1g) indicated the involvement of transcription factors in NIC-mediated increased EZH2 expression. EZH2 is regulated by molecules such as Myc, E2F, Sox4, Elk1, HIF1, etc.^34^. Apart from directly binding to E-box element of EZH2 promoter, Myc also regulates EZH2 expression by epigenetically repressing^35, 36^ the negative regulators miRNA-101 and miRNA-26a^37, 38^. miRNA-101 is reduced upon NIC treatment^39^. In a transcriptome study, Myc was found to be upregulated upon NIC treatment^40^.

To investigate the correlation between EZH2 and Myc, we analyzed MERAV gene expression dataset that is designed to analyze human gene expression across large variety of arrays (4454 arrays). The Pearson correlation coefficient values computed using its breast cancer cell line and patient sample datasets are 0.02 and 0.22, respectively (Fig. 5a, b). The values indicate that Myc and EZH2 shares a positive correlation, which is not very strong. The possible reason for which may be that only one transcription factor Myc may not be sufficient to regulate EZH2 in different heterogeneous tumors/cell lines with a diverse genetic background. The genetic diversity might be playing a role in regulation of EZH2 in association with Myc. Bupropion treatment resulted into reduced α9-nAChR, Myc, and EZH2 expression, indicating receptor-mediated upregulation of Myc and EZH2 in response to NIC treatment.

EZH2 expression is associated with tumor grade and aggressiveness of the disease. Higher is the tumor grade, more is the expression of EZH2. More aggressive is the disease, higher is the EZH2 expression. We were unfortunate to get the samples from lower grade of tumor such as grade 1 where probably the difference could be more distinct between smoking and non-smoking samples; however, even in higher grade of tumors, a notable difference was found in the EZH2 expression irrespective of the tumor grade and histological difference. The samples under study majorly display alternate combinations of breast ductal adenocarcinoma and lobular adenocarcinoma among never-smoked and smoking-associated breast cancer patients. Furthermore, the samples from never-smoked patients harbor low level of EZH2 irrespective of ductal or lobular type of cancer tissue in comparison to smoking-associated breast cancer tissue samples, which minimizes the role of inter-tumoral heterogeneity in EZH2 expression level.

In vitro data indicated increased EZH2 as a consequence of NIC treatment, which was strengthened by analyzing the effect of NIC post-DZNepA and EZH2si action. NIC is well studied for its immune suppressive role^41–44^ in humans. NIC and DZNepA treatment did not have significant effect on immune cell numbers in nude mice (Figure S1) as observed in Exigo veterinary hematology system. This suggested that the dosage used in the study for NIC and DZNepA did not affect the immune cells number. The body weights of the mice were found to be unaffected in the treatment groups, which also indicated that treatments did not have any adverse effect on the health of the animal.

The anatomical location has a significant influence on overall xenograft success^45^. The most appropriate site routinely used for breast tumor xenografts is mammary fat pad. We chose xenograft nude mice model to study the effect of NIC on tumor aggressiveness/implantation by modulating EZH2 expression and role of DZNepA in reducing NIC-induced increased tumor burden. One hundred percent tumor uptake is reported with MDA-MB-231 in the mammary fat pad (m.f.p.) injections whereas only 40% of the subcutaneous injections are reported to produce tumors several weeks after the appearance of the m.f.p. tumors^46, 47^. In our study, among 12 vehicle-treated mice, 4 (about 33.33%) showed up with tumors initially (Figure S3 showing the picture of three mice with a tumor size less than 100 mm^3^), but with time in only one mice, tumor of proper size (100 mm^3^) was successfully established and grew slowly compared to NIC-treated groups. In literature, different immune-deficient mouse strains have been used for establishing experimental tumors; however, in many instances their genetic background and source have not been clearly mentioned. Studies have evidently shown a distinct variability in establishing xenografts in different strains of immune-deficient mouse and this could be due to their differential residual adaptive and innate immunity present in these mice^48^. At the same time, the unavoidable variation in nude mice rearing and handling in different animal facilities might also contribute to the overall residual immune status of these mice. Taken together, we believe that the low percentage of tumor uptake in the vehicle-treated group of animals might be due to any of these possibilities.

Pierce et al. (2013)^40^ showed breast cancer deaths, recurrence, and survival proportions in never-smokers, former smokers, and current smokers, providing evidence to support NIC as a potent risk factor for breast cancer. Increased breast cancer deaths, recurrence, and reduced survival of current smokers indicate the role of NIC the most common constituent of cigarette smoke in providing survival advantage to the cancer cells. In support of the above study, our xenograft model showed higher uptake of tumor (higher number of xenografts successfully established) in NIC-treated animals in contrast to vehicle-treated mice, which corroborated with our in vitro data, suggesting that NIC provides survival advantage to the cancer cells by increasing the rate of proliferation (higher Ki67 positive, high MTT reading in vitro). Increased MTT reading and upregulated Ki67 and survivin expression upon NIC treatment suggest the role of NIC in increased successful tumor implantation.

Treatment with DZNepA in NIC-stimulated tumors resulted in reduced tumor growth, which shows its efficacy as a therapeutic drug in breast cancer treatment. DZNepA is an adenosine homocysteine hydrolase inhibitor that inhibits methyltransferases indirectly^49^. The mechanism underlying the inhibition of EZH2 by DZNepA communicates the involvement of other methyltransferases in NIC-mediated increased breast cancer risk, which needs further investigation. In conclusion, we demonstrate that in response to NIC there is an enhanced expression of Myc, which in turn increases EZH2 expression and results into increased breast cancer progression. Thus, polycomb group protein EZH2 plays a significant role in NIC-mediated increased breast cancer progression.

## Materials and methods

### Human breast cancer patient samples

Human breast carcinoma frozen tumor tissue sections from smoking and never-smoked female breast cancer patients used in the study were purchased from a US based company OriGene. Table S1 contains all the details of the patient samples used in the study. Breast cancer patient samples associated with smoking were matched with never-smoked ones depending on age, minimum stage of the disease, and Nottingham tumor grade system.

### Cell culture and treatment

The human breast carcinoma cell lines MCF-7, MDA-MB-231, T47D, and MDA-MB-453 were obtained from National Repository of Animal Cell Culture, NCCS Pune (Maharashtra, India) and independently validated by STR DNA fingerprinting at Institute of Life Sciences (Bhubaneswar, India). T47D, MDA-MB-231, and MDA-MB-453 cell lines were maintained in Roswell Park Memorial Institute 1640 (RPMI) medium and MCF-7 was maintained in Dulbecco’s modified Eagle’s medium (DMEM) containing 10% fetal bovine serum supplemented with penicillin–streptomycin at 37 °C, 5% CO_2_, and 95% humidity. MCF-10A, a kind gift from Dr. Annapoorni Rangarajan (IISC, Bangalore, India), was maintained in DMEM F12 containing horse serum (5%) supplemented with hydrocortisone (0.5 mg/ml), EGF (20 ng/ml), insulin (10 µg/ml), cholera toxin (100 ng/ml), and penicillin–streptomycin at 37 °C, 5% CO_2_, and 95% humidity. For treatment, appropriate concentration of DZNepA or NIC was directly added to the culture dish.

### EZH2 promoter assay

EZH2 promoter (1884 bp) was cloned into pGL3 luciferase vector (Addgene) using specific primers (Table S3). The generated pGL3EZH2 construct was transfected into MDA-MB-231 cells using Lipofectamine 3000 (Invitrogen) as per the manufacturer’s protocol. After 24 h of transfection NIC was treated and further after 48 h luciferase activity was quantified using Lucifease assay reagent (Promega). The luciferase reading was taken in a luminometer (Sirius, Titertek-Berthold).

### In vivo xenograft studies and drug administration

Institutional Animal Ethical Committee (Institute of Life Sciences, Bhubaneswar, India) approved all animal experiments. Five- to six-week-old BALB/c female nude mice (model NCRNU-F; nomenclature, CrTac: NCr-Foxn1^nu^; genotype-homozygous sp/sp) were used for xenograft study. MDA-MB-231 (10^7^) cells in matrigel were subcutaneously injected into the flank of the mice. After 3 days of cancer cell injection, intraperitoneal injection of NIC (0.25 mg/kg body weight) or PBS as a vehicle was administered twice in a week. When tumor size was about 400 mm^3^ in the NIC-treated group, the animals were randomly divided into NIC (0.25 mg/kg body weight) or NIC (0.25 mg/kg body weight) + DZNepA (1 mg/kg body weight) group and both the groups continued to receive the above-mentioned dose of NIC twice a week. Vehicle/DZNepA was treated every fourth day by intraperitoneal injection. The initial tumor size of both the groups before DZNepA treatment was around 400 mm^3^ as indicated in the graph (Fig. 3e). Experiment was terminated after 11 weeks when tumor in DZNepA-treated mice was ⩽100 mm^3^. Tumor volume was measured every fourth day with a digital caliper. The formula for calculation was tumor volume = 1/2(length × width^2^)^50^.

### Tissue processing and immunohistochemistry

Tissues were collected and preserved in 10% formalin. For histopathological analysis, tissues were processed for slide preparation. Immunohistochemistry in slides of both patient samples and xenografts was performed as described^51^. Slides were incubated with primary antibodies EZH2 (1:100) or Ki67 (1:100) overnight at 4 °C and then subjected to incubation with anti-mouse/rabbit IgG secondary antibody for 1 h. Diaminobenzidine was used to detect the immunoreactivity. Slides were subsequently stained with hematoxylin and processed further. Stained slides were observed under a light microscope (Leica DM500) and images were captured at ×40 magnification. Pathologist scored all the stained slides as previously described^52^. Briefly, the staining intensity of cancerous cells was scored as: absent or weak, 1 point; moderate, 2 points; and strong, 3 points. Percentage positive tumor cells were scored as: 0 for percent of cells <1, 1 for percent of cells between 1 and 10, 2 for percent of cells between 11 and 33, 3 for percent of cells between 34 and 66, and 4 for percent of cells between 67 and 100. *Q* score was calculated by multiplying intensity score by the score for percentage of EZH2-positive cancer cells.

### Cell viability assay

For cell viability assay, breast cancer cells were seeded at a density of 3 × 10^3^ cells per well in 96-well plates. NIC and/or DZNepA were added at appropriate concentrations directly into the medium after 12 h of cell attachment. Cells were incubated for desired time periods. After stipulated time, cell viability was checked by performing 3-(4,5-dimethylthiazol-2-yl)-2,5-diphenyltetrazolium bromide (MTT) assay^53^. The plate was read at a wavelength of 570 nm in a Varioskan™ Flash Multimode Reader (Thermo Scientific).

### Migration and invasion assays

Quantitative functional cell migration and invasion assays were performed using Boyden’s chambers consisting of polycarbonate filters with a pore size of 8 µm as previously described^54^. Appropriately NIC (10 µM, 120 h) or DZNepA (1 µM, 120 h) treated or siRNA transfected MDA-MB-231 cells were processed for the assay. After 24 h, cells migrated to the lower surface were fixed with 10% formalin and stained with 0.01% crystal violet stain. After washing, cells migrated towards the serum were observed under a Leica inverted microscope (ICC50 HD), pictured (×20) and counted in 10 different random field views. For invasion assay, the upper chamber of transwell was coated with a final concentration of 1 mg/ml of matrigel in serum-free growth medium.

### Western blot

Appropriately treated cells or xenografts were lysed in RIPA lysis buffer [20 mM Tris-HCl (pH 7.5) 150 mM NaCl, 1 mM Na_2_EDTA, 1 mM EGTA, 1% triton X, 1% sodium deoxycholate, 2.5 mM sodium pyrophosphate, 1 mM β-glycerophosphate, 1 mM Na_3_VO_4_, and 1 µg/ml protease inhibitor] and electrophoresed on 10% SDS-polyacrylamide gel. The proteins were transferred onto polyvinylidene difluoride (PVDF) membrane. After blocking in 5% skimmed milk the membrane was incubated with primary antibodies overnight (details of all antibodies and reagents are provided in Table S2). The membrane was then washed with TBS-T and incubated with anti-rabbit or anti-mouse horseradish peroxidase conjugated secondary antibody for 1 h. After washing, the blots were developed using luminol in Chemi-doc (Bio-Rad).

### Real-time PCR

Total RNA from treated breast cancer cells was extracted with Trizol as previously described^55^. Equal amount (1 µg) of RNA was used to synthesize cDNA of respective treated samples using cDNA synthesis kit following the manufacturer’s protocol. Quantitative real-time PCR was performed on the Roche platform. Details of primers are provided in Table S3. The relative mRNA level or fold change for each gene compared to control was calculated using the value of cycle threshold. Glyceraldehyde 3-phosphate dehydrogenase was used for normalization.

### siRNA transfection

For knockdown experiments, siRNA transfection was done using Lipofectamine 3000 transfection reagent according to the manufacturer’s instructions in appropriately treated MDA-MB-231 breast cancer cells. Sequence of siRNA duplexes is provided in Table S4. All experiments involving EZH2si transfection were done within 72 h.

### Annexin V apoptosis assay

Appropriately treated MDA-MB-231 (2 × 10^5^) cells were processed for apoptosis study using the Annexin V binding assay as per the manufacturer’s instructions. The cells were then analyzed using the FACS system (Becton Dickinson, USA).

### Online dataset

To investigate the correlation between EZH2 and MYC, we used online Metabolic gEne RApid Visualizer (MERAV) database. MERAV is designed to analyze normalized human gene expression across 4454 arrays. Our study includes 196 different established and patient derived breast cancer cell line and 332 primary breast tumors of different grades and histology types available in the dataset.

### Chromatin immunoprecipitation

ChIP assays were performed manually as per the cold spring harbor protocol^56^. Equal amounts of crosslinked DNA were used for Myc or control rabbit IgG. Input and immunoprecipitated DNA was purified using phenol-chloroform isoamyl alcohol and ethanol as described^57^. All samples were analyzed by qRT-PCR and were carried in duplicate with primers specific (Table S3) to Myc-binding region (E-box element) on EZH2 promoter. The fold enrichment was determined using the formula: Fold enrichment = 2^(ΔCT of input−ΔCT of Immunoprecipitated DNA)^^56^.

### Statistical analyses

Throughout the current study, two-tailed paired Student's *t*-test, one-way and two-way ANOVA was performed to test the statistical significance of differences between the experimental groups using the software GraphPad Prism v5.01. Differences in data with values of *P < 0.05, **P < 0.005 and ***P < 0.001 were considered statistically significant.

## Electronic supplementary material

Supplementary Data
