# Supplementary material for: Nicotine associated breast cancer in smokers is mediated through high level of EZH2 expression which can be reversed by methyltransferase inhibitor DZNepA
Source: Cell Death Dis. 2018 Feb 2;9(2):152. doi: 10.1038/s41419-017-0224-z (PMC5833686; doi:10.1038/s41419-017-0224-z)
Supplement: Supplementary file 1 — Supplementary Data [file 41419_2017_224_MOESM1_ESM.pdf]

Table S1: Breast Cancer Patient sample details

Sample	Currently smoking? (Week of surgery)	History of smoking?	Smoking history (details from medical record)	TISSUE ORIGIN	SITE OF FINDING	SAMPLE DIAGNOSIS	AGE	GENDER	TUMOR GRADE	MINIMUM STAGE GROUPING
a		Never smoked		Breast	Breast	Adenocarcinoma of breast, ductal	54	Female	NSABP Grade: Nuclear 2 of 3 & Histologic 2 of 3	IIIA
b	No (quit >1 yr prior surgery)	Yes	Patient does not smoke, having quit 25 years ago, prior to that she smoked 2 packs per day for 35 years.	Breast	Breast	Adenocarcinoma of breast, lobular	49	Female	Nottingham G2: 6-7 points Intermediate combined grade (moderately favorable)	IIIA
c	No (never smoked)	Never smoked	No history of tobacco use.	Breast	Lymph node	Adenocarcinoma of breast, lobular, metastatic	54	Female	Nottingham G3: 8-9 points High combined grade (unfavorable)	IIIC
d	No	Yes	Patient smoked 1 pack per day for 35 years but quit 6 months to 1.5 years prior to surgery, entered average of 1 year quit	Breast	Breast	Adenocarcinoma of breast, ductal	58	Female	Nottingham G3: 8-9 points High combined grade (unfavorable)	IV
e		Never smoked		Breast	Breast	Adenocarcinoma of breast, ductal, metastatic	64	Female	Nottingham G3: 8-9 points High combined grade (unfavorable)	IIIA
f		Yes	quit tobacco four weeks ago and reports a 20-pack-year smoking history	Breast	Breast	Adenocarcinoma of breast, ductal	66	Female	Nottingham G2: 6-7 points Intermediate combined grade (moderately favorable)	IIIA
g		Never smoked		Breast	Breast	Carcinoma of breast, squamous cell	54	Female	Nottingham G3: 8-9 points High combined grade (unfavorable)	IIB
h	No (quit >1 yr prior surgery)	Yes	Smoked for 3 years as a teenager, but does not currently use tobacco. [Note: Quit approximately 28 years ago.]	Breast	Breast	Adenocarcinoma of breast, ductal (triple negative)	47	Female	Nottingham G3: 8-9 points High combined grade (unfavorable)	IIB
i	No (never smoked)	Never smoked	No history of tobacco use.	Breast	Lymph node	Adenocarcinoma of breast, lobular, metastatic	54	Female	Not Reported	IIIC
j	No (quit >1 yr prior surgery)	Yes	Smoked one pack per day for five years	Breast, left	Breast, left	Adenocarcinoma of breast, ductal	56	Female	NSABP Grade: Nuclear 3 of 3 & Histologic 3 of 3	IIIC
k	No (never smoked)	Never smoked	Patient has never smoked cigarettes.	Breast	Breast	Adenocarcinoma of breast, ductal	46	Female	Nottingham G3: 8-9 points High combined grade (unfavorable)	IIA
l	No (quit >1 yr prior surgery)	Yes	Smoked 4 packs a year "in college."	Breast	Breast	Adenocarcinoma of breast, ductal	68	Female	Nottingham G3: 8-9 points High combined grade (unfavorable)	IIA

Table S2: Details of antibodies and reagent

Antibody/Reagent/Kit	Catalog no.	Company
anti-EZH2 antibody	D2C9-5246	Cell Signaling Technology
anti-nAChRα9	sc-13806	Santa Cruz Biotechnology, INC
anti-cytokeratin14	sc-53253	Santa Cruz Biotechnology, INC
anti- fibronectin	sc-18825	Santa Cruz Biotechnology, INC
α-tubulin antibody	T5168	Sigma Aldrich, USA
anti-Rabbit IgG (whole molecule)–Peroxidase antibody produced in goat secondary antibody	A0545	Sigma Aldrich, USA
anti-mouse HRP conjugated secondary antibody	A3682-1ML	Sigma Aldrich, USA
EMT antibody sampler kit	9782S	Cell Signaling Technology
4, 5-dimethylthiazol-2yl)-2, 5-diphenyl tetrazolium bromide (MTT)	2102227.1	MP Biomedical, USA
PE Annexin V Apoptosis detection kit	559763	BD Biosciences
SYBER Green	RT-SY2X-03+NRWOU	Eurogentech
Nicotine	N3876	Sigma Aldrich, USA
3-Deazaneplanocin A hydrochloride	SML0305	Sigma Aldrich, USA
SuperScript® First-Strand Synthesis System for RT-PCR	11904018	Invitrogen, Carlsbad, CA
Dnase I Kit	AMPD1-1KT	Sigma Aldrich, USA
INTERFERin Polyplus-Transfection reagent	409-10	Polyplus-transfection® SA
Lipofectamine 3000	L3000001	Invitogen
PVDF membrane	88518, 0.45µm,	Thermo scientific
Skimmed milk	RM1254-500G	Himedia
ECL-HRP for X-ray Film Kit	K-12045-D50	Advansta
Boyden's chambers (0.8µm)	353182	BD Falcon, USA
Matrigel	356237	BD Pharmingen, USA
Trizol	T9424	Sigma Aldrich, USA

Table S3: Primer sequence

S. No.	Primer name	Oligo Sequence 5' to 3'
1	SNAI1.RT.F	GGACTCTAATCCAGAGTTTACC
2	SNAI1.RT.R	CAGAGTCCCAGATGAGCATT
3	SNAI2.RT.F	CTGGACACACATACAGTGATT
4	SNAI2.RT.R	GGAGTATCCGGAAAGAGGAG
5	ZEB1.RT.F	CTCATTGTGGAGAGATGACT
6	ZEB1.RT.R	ATAATTTGTAACTTTCATAAAGCC
7	NCAD.RT.F	GGCAGTAAAATTGAGCCTGA
8	NCAD.RT.R	TCAGACCTGATCCTGACAAG
9	CDH1.RT.F	CGCGTCCTGGGCAGAG
10	CDH1.RT.R	ACGGAGGCCTGATGGG
11	VIM.RT.F	CGAGGAGAGCAGGATTTCT
12	VIM.RT.R	GTGATGCTGAGAAGTTTCGT
13	FN1.RT.F	CACTCTCGGAATTCCATCAC
14	FN1.RT.R	GGCTATTTCCTCCTGTCTCT
15	Ecad.RT.F	GGTGCTCTTCCAGGAACCTC
16	Ecad.RT.R	GGAAACTCTCTCGGTCCAGC
17	5'Ex1Ezh2	AGAAGGGACCAGTTTGTTGG
18	3'Ex1Ezh2	TTCATCAGCTCGTCTGAACC
19	Ezh2 Promo. F1 XhoI	GACCTCGAGAGTGTGAGACTCCGTTT
20	Ezh2 Promo. R1 HindIII	CTCAAGCTTCACTGCCTTCTGAGTC
21	EZH2 E-box_F	CCTTGCAAATATCCTCACACTT
22	EZH2 E-box_R	GTTTGATTATGTCTGCTGCTG

Table S4: siRNA Duplexes

**Pooled EZH2si Duplexes (Eurogentech)**
5'-GGG-AAA-GUG-UAU-GAU-AAA-U55-3'
5'-AUU-UAU-CAU-ACA-CUU-UCC-C55-3'
5'-CAC-AAG-UCA-UCC-CAU-UAA-A55-3'
5'-UUU-AAU-GGG-AUG-ACU-UGU-G55-3'
5'-GGA-UGG-UAC-UUU-CAU-UGA-A55-3'
5'-UUC-AAU-GAA-AGU-ACC-AUC-C55-3'

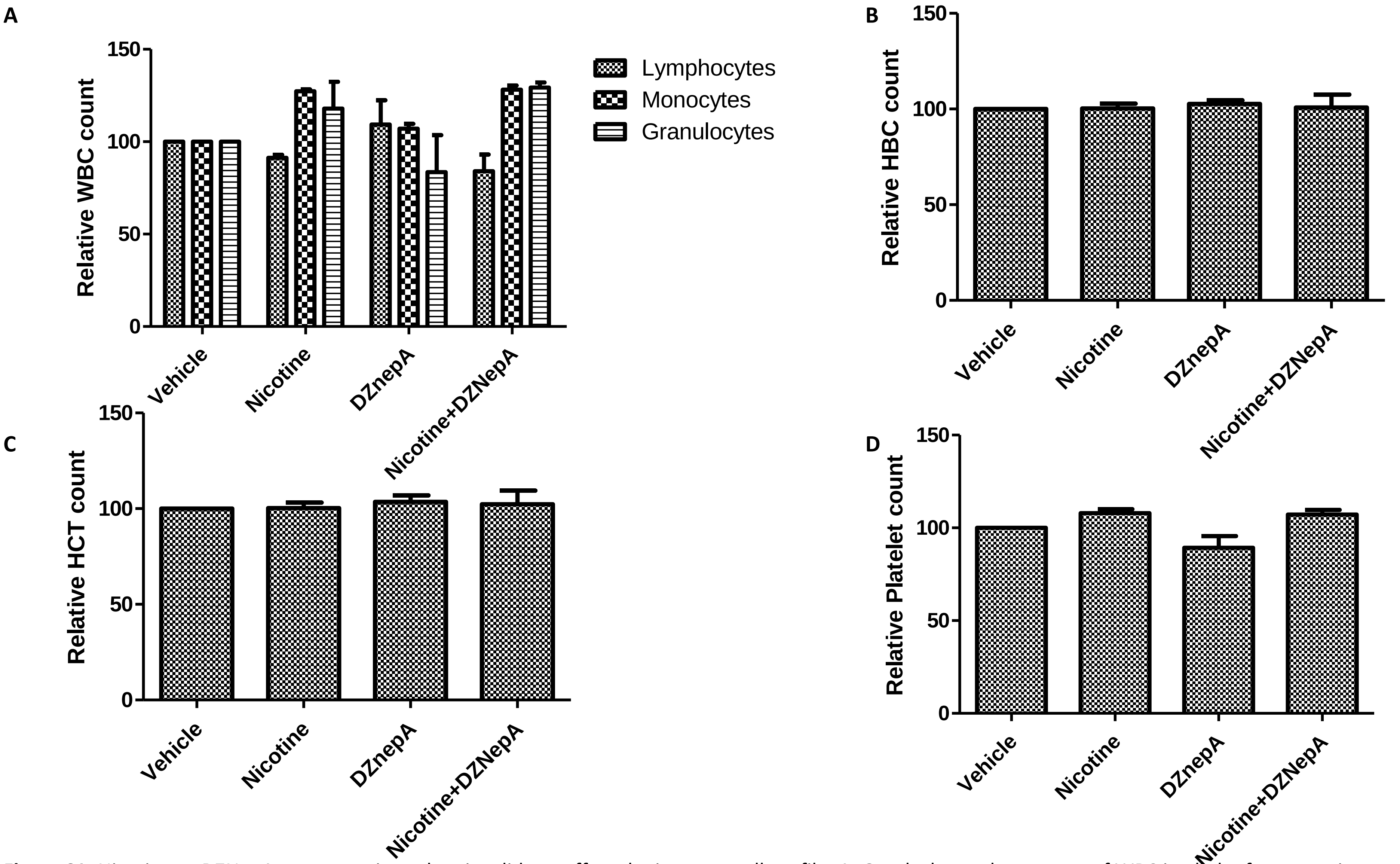

Figure S1: Nicotine or DZNepA treatment in nude mice did not affect the immune cell profile. A. Graph shows the percent of WBC in all the four experimental groups. B, C and D. Graphical representation of hemoglobin, haematocrit and platelet count respectively in vehicle, nicotine, DZNepA and nicotine & DZNepA co-treated mice. The results are shown from treatment done in triplicate.

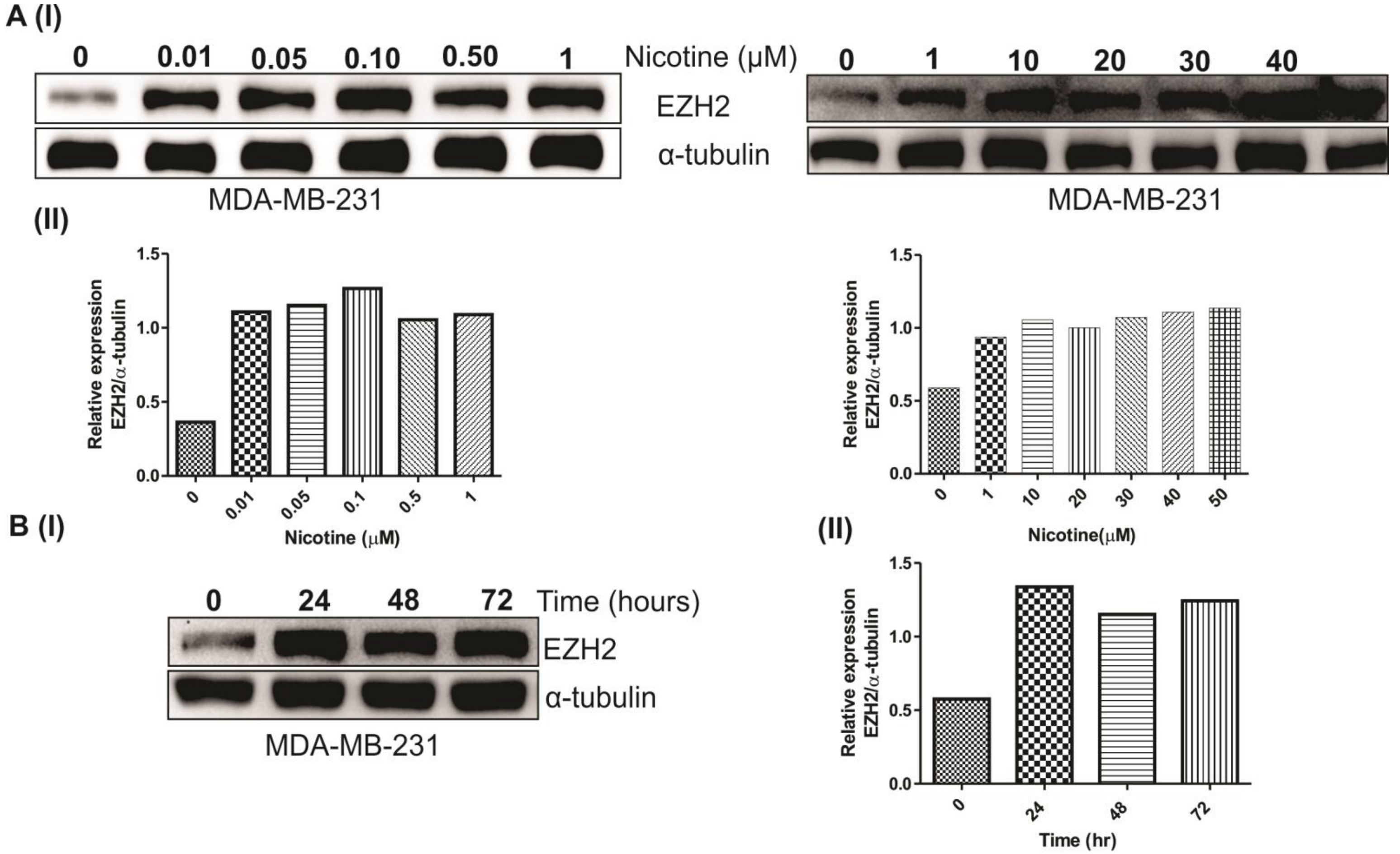

Figure S2: AI and B I, EZH2 expression at different concentration of nicotine and at different time intervals respectively was examined using western blot assay. A II and B II, Graphs represents the respective normalized EZH2 expression in the immunoblot.

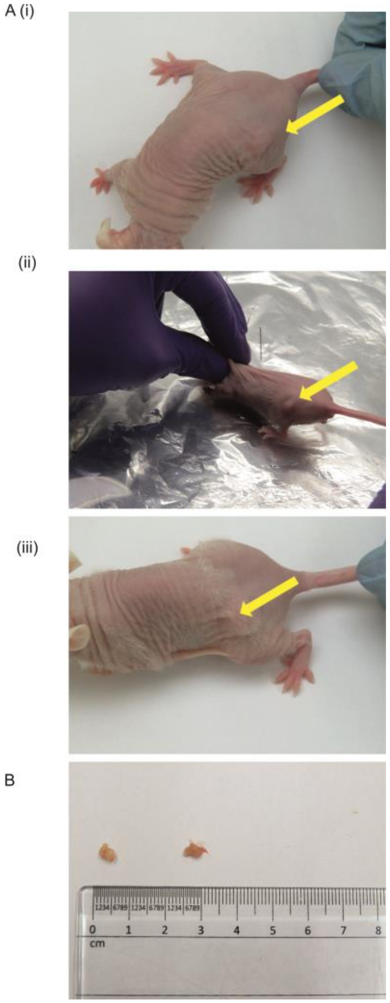

Figure S3: A (i), (ii), (iii) Pictures of vehicle treated mice with tumor size less than $100mm^3$. B. Picture showing the tumors collected from vehicle treated mice (A(i) and (ii)). There was no palpable and visible tumor of mice A (iii) (regressed completely) at the end of the experiment. Arrow indicates the site of tumor implantation.
